# Editorial: Can't Get You Out of My Head: Brain-Body Interactions in Perseverative Cognition

**DOI:** 10.3389/fnhum.2017.00634

**Published:** 2017-12-19

**Authors:** Cristina Ottaviani, Julian F. Thayer, Bart Verkuil, Hugo D. Critchley, Jos F. Brosschot

**Affiliations:** ^1^Department of Psychology, Sapienza University of Rome, Rome, Italy; ^2^Neuroimaging Laboratory, IRCCS Santa Lucia Foundation, Rome, Italy; ^3^Department of Psychology, The Ohio State University, Columbus, OH, United States; ^4^Clinical Psychology Unit, Institute of Psychology, Leiden University, Leiden, Netherlands; ^5^Psychiatry, BSMS Department of Neuroscience, Brighton and Sussex Medical School (BSMS), University of Sussex, Falmer, United Kingdom; ^6^Sackler Centre for Consciousness Science, University of Sussex, Falmer, United Kingdom; ^7^Health, Medical, and NeuroPsychology Unit, Institute of Psychology, Leiden University, Leiden, Netherlands

**Keywords:** perseverative cognition, functional connectivity, heart rate variability, brain-body interaction, generalized anxiety disorder

Perseverative cognition represents a prototypical example of how our internal thoughts can impact our psychological and physical health, as if we were facing an actual environmental stressor (Brosschot et al., 2006). The mechanisms involved—together with other emblematic examples like the placebo effect—provide clear evidence for brain-body interaction. This collection of articles presents recent advances in our understanding of perseverative cognition that have arisen from the integration of multidisciplinary approaches encompassing cognitive and clinical psychology, affective neuroscience, and autonomic physiology. These advances carry with them the promise of more effective treatments to mitigate the negative consequences of maladaptive perseverative cognition on health and well-being.

All contributions to the present Research Topic share a definition of perseverative cognition as a rigid pattern of habitual repetitive thoughts that perpetuates threat/stress responses through a characteristic failure of regulatory inhibition. Physiologically, perseverative cognition is expressed across multiple axes, including cardiovascular, autonomic, and endocrine systems (Ottaviani et al., 2016). Among physiological indices, heart rate variability (HRV) has emerged over the past decade as a biomarker that is particularly well-suited for indexing the inflexibility intrinsic to perseverative cognition. In fact, the adaptive rapid application and withdrawal of vagal parasympathetic inhibition, reflected in HRV, is viewed as a dynamic substrate for flexible behavioral routines (Porges, 2007).

The first group of papers precisely explores this link between perseverative cognition and HRV. Two papers in particular combine the measurement of autonomic inflexibility, indexed by HRV, with measures of attentional/cognitive rigidity that characterizes perseverative cognition: Gazzellini et al. reveal that individuals who are highly prone to worrisome thinking show increased variability in reaction times (following a periodic oscillating pattern), recurrent lapses in attention, and concomitant oscillating heart rate. The authors suggest that, at a central level, these predictable fluctuations are mediated by midline cortical structures belonging to the default mode network. Spangler and Friedman provide relevant evidence for cardiac vagal control as an index of the availability of working memory resources: Anxiety impairs one's ability to focus attention and inhibit distractors. Minimal working memory load can attenuate this detrimental effect of anxiety, but heavier working memory demands may compromise attentional inhibition to the same extent as anxiety itself. Cropley et al. leave the laboratory setting in pursuit of a more ecological evaluation of perseverative cognition and HRV. This combined ambulatory assessment, over three consecutive evenings, provides the first evidence that individuals who have a tendency to ruminate about work also have lower HRV after work, when compared to individuals who report low levels of work-related rumination.

The contribution of Williams et al. takes a fairly different perspective in which HRV is considered as a trigger, rather than a consequence, of perseverative cognition. This is based on the assumption that individuals with lower resting HRV are more vulnerable to stress, and therefore are more likely to engage in maladaptive types of perseverative cognition (e.g., brooding rumination). Moreover, an indirect effect of maladaptive perseverative cognition on resting HRV can feedback to make these same individuals more susceptible to anxiety symptoms. Notably, adaptive types of rumination (i.e., reflective rumination) do not significantly mediate this association, suggesting that not all rumination is pathogenic. The paper by Diamond and Fisher applies similar notions to major depressive disorder, generalized anxiety disorder (GAD), and social anxiety disorder by exploring autonomic stress responses to a clinical diagnostic interview in these clinical populations and matched controls. The groups showing the highest autonomic rigidity during the interview were high-worriers, and patients with GAD, who have perseverative cognition as diagnostic requirement. The authors conclude that shared transdiagnostic features, notably worry and suppression, rather than diagnostic comorbidities, account for physiological reactivity across patient groups.

It is important to note that the pathogenic effects of rumination can be partially explained by its capacity to engender behaviors that put health at risk (substance use, alcohol consumption, unhealthy eating, and smoking), as shown by the meta-analysis conducted by Clancy et al. Surprisingly, this route could not be demonstrated for worry. This contribution elegantly illustrates that another plausible route linking rumination to disease is via poorer health behaviors.

Both Toh and Vasey and Meeten et al. provide unique information about the processes underpinning the conceptualization of worry as a strategy of cognitive avoidance in response to threat, via its verbal nature (Borkovec et al., 2004). The contribution by Toh and Vasey clarifies that worry predicts lower autonomic arousal, but only at high levels of effortful control that are characterized by a greater emphasis on verbal thoughts. Meeten et al. further explore the neurobiological correlates of the Borkovec's Cognitive Avoidance Model and theoretical mechanisms of perseveration (i.e., the tendency to deploy goal-directed worry rules; Davey and Meeten, 2016). The authors used a perseverative cognition induction in patients with GAD and controls to quantify decreases in HRV and associated changes in patterns of functional connectivity of the amygdala that provide insight into the biological processes underlying explanatory models of worry.

The Research Topic ends with a contribution that directly addresses treatment interventions: Fresco et al. used resting state functional magnetic resonance imaging before Emotion Regulation Therapy (Fresco et al., 2013) in GAD and tested if functional connectivity patterns predict subsequent treatment-related changes. The authors first confirmed previously reported disruptions in the default mode and salience networks in GAD. Notably, functional connectivity patterns within these networks were associated with treatment-related changes in worry, somatic anxiety, and decentering.

Perseverative cognition remains difficult to treat, despite being a recognized risk factor for health and a transdiagnostic symptom that commonly occurs in everybody's life. Overall, the present Research Topic substantially enhances the understanding of the factors that trigger, maintain, and follow perseverative cognition. Importantly, these fresh insights occur through the perspective of brain-body interactions. The clarification of these factors is essential to inform therapeutic interventions that may take either bottom-up (parasympathetic-to-brain) or top-down (brain-to-parasympathetic) approaches.

## Author contributions

All authors (CO, JT, BV, HC, and JB) have contributed to this Editorial. CO has drafted the Editorial, JT, BV, HC, and JB provided intellectual contributions in commenting and revising the manuscript.

### Conflict of interest statement

The authors declare that the research was conducted in the absence of any commercial or financial relationships that could be construed as a potential conflict of interest.
